# Undergraduate Medical Education Research in Malaysia: Time for a Change

**DOI:** 10.12669/pjms.313.7389

**Published:** 2015

**Authors:** Abdus Salam, Jemaima Che Hamzah, Tan Geok Chin, Harlina Halizah Siraj, Ruszymah Idrus, Nabishah Mohamad, Azman Ali Raymond

**Affiliations:** 1 Abdus Salam, MBBS; MPH; PgDipMedEd; MMedEd. Associate Professor, Department of Medical Education, Universiti Kebangsaan Malaysia (UKM) Medical Centre, Faculty of Medicine, Kuala Lumpur, Malaysia; 2 Jemaima Che Hamzah, MD; MS Opthal; PhD. Associate Professor, Department of Ophthalmology, Universiti Kebangsaan Malaysia (UKM) Medical Centre, Faculty of Medicine, Kuala Lumpur, Malaysia; 3 Tan Geok Chin, MBBS; MPath; AM; PhD. Associate Professor, Department of Pathology, Universiti Kebangsaan Malaysia (UKM) Medical Centre, Faculty of Medicine, Kuala Lumpur, Malaysia; 4 Harlina Halizah Siraj, MD; MOG. Associate Professor, Department of Medical Education, Universiti Kebangsaan Malaysia (UKM) Medical Centre, Faculty of Medicine, Kuala Lumpur, Malaysia; 5 Ruszymah Idrus, MD; PhD. Professor, Department of Physiology, Universiti Kebangsaan Malaysia (UKM) Medical Centre, Faculty of Medicine, Kuala Lumpur, Malaysia; 6 Nabishah Mohamad, MD; PhD; PgDipMedEd. Professor of Physiology & Medical Edu and Deputy Dean (Undergraduate Studies and Alumni), Universiti Kebangsaan Malaysia (UKM) Medical Centre, Faculty of Medicine, Kuala Lumpur, Malaysia; 7 Azman Ali Raymond, MBBS Hons.; MMed; MMed; MD; AM; FRCP. Professor of Medicine, Dean, and Director, Universiti Kebangsaan Malaysia (UKM) Medical Centre, Faculty of Medicine, Kuala Lumpur, Malaysia

**Keywords:** Undergraduate, Medical education, Research, Publication, Malaysia

## Abstract

**Objective:**

Special Study Module (SSM) is a mandatory research module implemented in Universiti Kebangsaan Malaysia (UKM). The objective of this paper is to provide a brief overview on the student research activities and to find out the outcome measures in terms of publication.

**Methods:**

It was a retrospective study done on SSM research projects at UKM. The SSM research is conducted from beginning of year-4 until 1^st^ seven weeks of year-5. In year-4, students are assigned to a faculty-supervisor in small groups and spend every Thursday afternoon to plan and carry the research. Whole first seven weeks of year-5, students are placed with their supervisor continuously to collect data, do analysis, write report and present in the scientific conference. Outcomes of 5-years SSM research-projects starting from 2008/2009 to 2012/2013 academic session were analyzed.

**Results:**

Total 257 projects were completed and presented in annual scientific meetings from which 57 (22.2%) articles were published in peer reviewed journals.

**Conclusion:**

Mandatory undergraduate student research project brings an opportunity to develop students’ capacity building from conception to final report writing and thereby narrowing the gap between education and practice. Medical schools should implement research module to bring changes in research and publication culture of undergraduate medical education.

## INTRODUCTION

Research is an essential guide to improve and develop new inventions in the health system^1^ so that we are able to meet the challenges in the health system. There are constantly new discoveries and inventions in the medical sciences and doctors need to keep up to date with the recent developments to justify their clinical decision with established facts and evidences. Thus, student research during the clinical training enables them to learn about evidence based practice which is important to create a responsible best-informed doctor.^2^ It is mentioned that health research can be promoted by medical students during the earlier part of their career by involving them in research activity together with their training. Students with research experience during their undergraduate program are more involved in postgraduate research activities later in life.^3^ Today much emphasis has been given in many medical schools to stimulate student’s interest in research by undertaking a mandatory course on research which can allow them to carry out all the steps of a research project from conception to final report writing, thereby narrowing the gap between theory and practice.^4^ Undergraduate medical student research projects may provide an opportunity for students to learn research methodologies and skills for the critical analysis of published literature.^5^

Regarding student research activities in developing countries, there are reports that some research activities were carried out in India, Pakistan, Nepal and Croatia. In the developed country, the importance of the student research activities are recognized very well and it is incorporated in the curriculum.^6^ An extensive research on medical students showed that medical students with research experience have a positive impact and these programs encourage students to pursue their future research activities and enter academic and research career.^7^ Moreover, publishing the research activities in a journal is a realistic view of one’s ability that the work is well regarded by a disciplinary community. Students who publish during their studies are more able to publish after they graduate.^8^

Universiti Kebangsaan Malaysia (UKM) is the national university meeting the needs of its diverse student population. Undergraduate medical education in UKM comprises of varieties of teaching learning modalities and research is one of the important and compulsory modality in year 4 and year 5 medical curriculum. The special study module (SSM) is a research study program implemented in UKM, aimed to develop students’ research skill as well as interpersonal skill to produce an all rounded doctors who will be well versed in the research aspect as well. This SSM project enhances students soft skills such as communication skill, leadership, critical thinking, problem solving and team work. Students also learn to present their final report in a student scientific conference with an aim to publish their work in scientific journals. Therefore, the SSM project has given special emphasis and is a pre requisite for the final professional examination for the medical students in UKM.

The objective of this paper is to provide a brief overview on the student research activities undertaken by the UKM and to find out the total research projects and publications accomplished from these research activities. This paper may encourage other teaching institutes to include research activities in their undergraduate curriculum.

## METHODS

### SSM research project

The SSM research is conducted from the beginning of year 4 until 1^st^ seven weeks of year 5 of their academic sessions. All 4^th^ year students are divided into small groups and assigned with a lecturer as the supervisor who had research experience to guide them along the way. Usually there are 4-7 students in a group and they were given the opportunity to choose the type of research they are interested from different aspects of medicine ranging from clinical to laboratory health service or community based medicine. Thus students are assigned to the lecturers involving all the departments of the institute.

The SSM project comprised of three sub module namely (i) proposal development and submission, held in 1^st^ semester of year 4 of each academic session (ii) ethical approval and organization of data collection, held in 2^nd^ semester of year 4 of each academic session and (iii) data collection, analysis and report writing held in 1st semester of year 5 of each academic session. The SSM research projects started with the research planning process which included research design, literature review, development of the research proposal, and then submission of the research proposal through the respective department to the ethical committee of the UKM. Before submission, students need to present their proposal in the respective department where they are assigned to supervisor. All the departmental lecturers are usually present during the presentation. This presentation helped them to refine and review their proposal through the comments given by the attending lecturers. After getting approval from the ethical committee, the final work of the research such as actual research work, data collection, analysis, writing the final report and presentation of the final report to the department are completed. Throughout the whole year-4, students are allocated one afternoon per week i.e. every Thursday to do SSM research activities. During this time, students worked with their project and meet with their supervisor who guided them throughout the work. Students are also supported by the statistical expert for guidance and provided required budget for their research expenditure. During the whole first seven weeks of year 5, students are attached to their supervisor in a seven week SSM posting to do data collection, analysis, and report writing in the form of manuscript.

Assessments of the students are done after every sub module using a scoring rubric. Two types of assessment are used: group and individual. Group assessments are based on the proposal submission and final report by the supervisor as well as by at least two independent departmental lecturers who are not involved in the research during their presentation in the department. Individual assessments are based on supervisor report, peer evaluations and reflective writing on the research activities of the sub module. At the end of the SSM posting, the students organize a scientific conference titled “Medical Undergraduates Annual Scientific Research Meeting” (MUASRM) where students present all their research works. During this one-day event, dissemination of research findings is done through oral and poster presentation. The abstracts of all the research projects are published in the MUASRM abstract book. Prizes are awarded to top three best research presentations in both groups. The full manuscripts are then submitted by the respective SSM research groups to the different journals for publication.

### Study design

A retrospective study involving all SSM research projects compulsorily conducted by UKM medical students since its inception i.e. starting from 2008/2009 academic session were selected. Data regarding the total number of publications resulted from the SSM projects till December 2014 was retrieved from the secretariat of undergraduate studies and alumni. Compilation of five academic year SSM research projects and their publication status were documented and analyzed.

## RESULTS

Table-I showed the total number of SSM research projects accomplished and their publication status from 2010 to 2014. A total 257 projects were accomplished in five years from which 57 (22.2%) articles were published in peer reviewed journals. The year wise publication status of SSM research projects were also shown here in this table.

**Table-I:** Distribution of SSM research projects and publication accomplished in five years.

Academic Session	SSM Project Accomplished	Articles Published		Year of Publication
		No	Percent	
2008/2009	42	16	38.1	2010
2009/2010	42	9	21.4	2011
2010/2011	57	22	38.6	2012
2011/2012	57	8	14.0	2013
2012/2013	59	2	3.4	2014
5 Batches	257	57	22.2	5 Years

## DISCUSSION

The SSM is a research module that aspires to develop the scientific vision of the medical students which can help them in their future research activities. The SSM research project is one of the best ways to upgrade the undergraduate research activities in the university. This present study showed that a total of 257 students’ SSM research projects have been accomplished in five years period since its implementation in 2008/2009 and a total of 57 (22.2%) publications were already available in the national and international peer reviewed journals and some others are in the process of publication. Although not all the research reports were published, still the exposure of research activities to the students is still considered as beneficial.

In addition, 257 project abstracts were published in the MUASRM abstract books, where students presented their research findings. Through these annual scientific research meetings students are able to interact with their fellow colleagues and lecturers as well as exchange of innovative ideas and knowledge explored through research. This scientific meeting allows them to realize the vision of a research university. It is hoped that they will be more innovative and be able to conduct excellent scientific research work in future as a medical graduate. This will ultimately help to develop the research activity in Malaysia.

Previous study has shown that the writing skill and information retrieving skill were significantly higher for students in undergraduate research projects than for students in the basic skill electives or the lecture based course.^9^ It is reported that in a German medical faculty, students’ research activities significantly influence their publication. Twenty eight percent of the publications were authored by students and among them 7.8% were the first authors.^10^ One of the study focused on the perception, attitude and practice toward the research activity reported that majority believe research activity will help them in their education and 44% believe that it will help in their future career.^11^ This present study revealed that the publication status varied from a minimum of 3.4% to a maximum of 38.6% with an average of 23.1% during last 5 years (Table-I). Students were the authors in all of these publications where in some papers they were the first authors and in some papers as co-authors, as decided by the group members and supervisors. This finding has consistency with Cursiefen and Altunbas (1998). In terms of publication, it is necessary to give much emphasis in writing and publishing the work because it is not easy to publish scholarly manuscripts. It is mentioned in the literature that writing is considered as a hard work by some academics.^12^,^13^ Thus, writing with the collaboration of expert supervisor may be finally able to publish the work.

The student research activity can help to develop the skill in searching and critically appraising the medical literature and independent learning.^14^ Akman et al.(2010) reported that it is essential to start research education in early medical years in order to keep students interest on scientific research.^15^ Our research program is also a great achievement in view of the vision of the building of research universities. UKM being a research university are directed to upgrade research and development activities and this may trigger healthy competitive nature within the public universities that will increase quality and quantity of countries invention and innovation.^16^

In addition to our reported 257 SSM research projects for the period of 5 years starting from 2008/2009 to 2012/2013 academic session, 59 SSM research projects have been completed in academic session 2013/2014 and presented in the 6^th^ MUASRM in 2014 and are in the process of submissions to different journals for publication. In the current 2014/2015 academic session another 40 SSM projects are in the process which will present in 7^th^ MUASRM in 2015. It is expected that a number of publications will be increased more and more in due course of time in the national and international journals from these researches.

Though student research activities are included in many medical schools, there are some barriers in its implementation. Inadequate supervision is one of the main barriers. In Finland, lack of good supervisor was mentioned as one of the leading cause.^17^ Other causes include lack of time, neglect of routine studies, and deterioration of the clinical skill due to more time being spent on research activities, and inadequate project management.^18^ Availability of adequate fund is also a barrier particularly in the developing countries. Lack of interest, and inadequate knowledge in research activities or lack of good research proposals are also the common barrier for the developing countries.^1^ It is the responsibility of the university to update the curriculum and utilize and mobilize the proper resources in terms of time management and assigning the experienced dedicated supervisors to bring changes in the field of research and publication in undergraduate medical education.

## CONCLUSION

The outcome measures of UKM undergraduate medical students’ research projects in terms of publication though is not very high, it is not bad. The SSM research project shows a very potential prospect in establishing research and publication attitude among students and supervisors of UKM. Mandatory undergraduate student research project is an opportunity to develop students’ capacity building in research and thereby narrowing the gap between theory and practice. This definitely will increase the research outcomes and a substantial number of publications can be accomplished from the student research projects. Now is the time for change and medical schools should implement compulsory special study research module to bring changes in the research and publication culture of undergraduate medical education.
